# One way or another, you are not going to fit: trans and gender diverse people’s perspectives on sexual health services in the United Kingdom

**DOI:** 10.1136/sextrans-2024-056231

**Published:** 2025-01-20

**Authors:** Tom Witney, Greta Rait, John Saunders, Lorna Hobbs, Laura Mitchell, Jay Stewart, Lorraine K McDonagh

**Affiliations:** 1 Institute for Global Health, UCL, London, UK; 2 The National Institute for Health Research Health Protection Research Unit in Blood Borne and Sexually Transmitted Infections at University College London in Partnership with the UK Health Security Agency, London, UK; 3 Department of Primary Care and Population Health, UCL, London, UK; 4 Blood Safety, Hepatitis, STIs and HIV Division, UK Health Security Agency, London, UK; 5 Tavistock and Portman NHS Trust, London, UK; 6 University Hospitals Plymouth NHS Trust, Plymouth, UK; 7 Gendered Intelligence, London, UK

**Keywords:** Sexual and Gender Minorities, SEXUAL HEALTH, Patient Participation, HEALTH SERVICES RESEARCH, QUALITATIVE RESEARCH

## Abstract

**Abstract:**

**Objectives:**

Trans and/or gender diverse (T/GD) people in the UK are less likely to access sexual health services (SHS) than cisgender people and are more likely to report negative experiences. The British Association for Sexual Health and HIV (BASHH) developed expert recommendations for T/GD-inclusive SHS, but these lack service user perspectives. This study addressed this gap by asking T/GD people how SHS could be T/GD-inclusive.

**Methods:**

Semistructured interviews (n=33) and focus groups (n=26) were conducted with T/GD people aged 17–71 years old recruited through community organisations and social media, exploring experiences of SHS and inclusivity. Study design, materials and analysis were informed by T/GD people and an advisory committee of charities and sexual health clinicians. Data were analysed using thematic analysis, managed using NVivo.

**Results:**

Participants often expected that SHS were not set up for T/GD people. This was reinforced by poor experiences in other healthcare settings and the lack of information on NHS websites. Some participants had been denied care because they were ‘too complex’. Participants wanted to know that SHS had engaged with the needs of T/GD people and looked for hallmarks of inclusivity, such as Trans Pride flags in reception areas. Some participants wanted specialist T/GD services, but others preferred to access general SHS. Staff attitudes were a key factor underpinning inclusivity. Anticipating having their identity questioned or needs dismissed, participants sought kindness and openness. Although the needs of T/GD people are diverse and different from cisgender service users, participants stressed that SHS staff already had the skills to deliver sensitive person-centred care and emphasised the value of inclusive SHS.

**Conclusion:**

These findings provide insight into what a sample of T/GD people in the UK consider important for T/GD-inclusive SHS. Participants’ suggestions align with and reinforce BASHH expert recommendations. Importantly, they highlight the need for ongoing engagement to deliver T/GD-inclusive SHS.

**WHAT IS ALREADY KNOWN ON THIS TOPIC**

**WHAT THIS STUDY ADDS**

- Participant suggestions and preferences for inclusive services support BASHH GSM recommendations.
- Participants anticipated that SHS services were unlikely to meet their needs and looked for signs that services were actively engaged with T/GD inclusivity.

**HOW THIS STUDY MIGHT AFFECT RESEARCH, PRACTICE OR POLICY**

- Enhancing T/GD inclusivity requires ongoing engagement with BASHH expert recommendations, which encompass clinical spaces, processes and consultations.
- Involving T/GD people in the development of services can enhance and strengthen recommendations.

## Introduction

 Transgender is an umbrella term to describe people whose gender identity differs from their sex assigned at birth. Gender diversity refers to the extent to which an individual’s gender identity, role or expression differs from the cultural norms prescribed for a particular sex. This includes those who identify as transgender and those who do not identify within the traditional gender binary.^1^ Robust estimates of the number of trans and/or gender diverse (T/GD) people in the UK are lacking, however, the 2021 Census for England and Wales included optional questions about gender identity for the first time and 262 000 (0.5%) respondents indicated their gender was different to sex assigned at birth.^2^ The number of people reporting a transgender identity in UK primary care records has increased fivefold from 2000 to 2018.^3^

T/GD people in the UK face high levels of violence, harassment, stigma, discrimination, marginalisation, homelessness and underemployment.^4^,^6^ These are associated with increased rates of alcohol and substance use, anxiety, depression and suicidal ideation compared with cisgender people.^7 8^ T/GD people face multiple, intersecting barriers to accessing healthcare across diverse settings.^9^,^11^ Lack of information about sexual and reproductive health needs of T/GD people, among both service users and providers, is a barrier to good quality care.^12^ A survey of T/GD people in the UK suggested they are less likely to use sexual health services (SHS) than cisgender people.^13^ T/GD people using online sexual health testing had higher rates of HIV and other STIs compared with cisgender service users and reported potentially complex sexual health needs.^14^ However, HIV prevalence data are inconsistent; the first population estimate of HIV prevalence among T/GD people in England in 2021 suggested a similar prevalence to the general population.^15^

In response to lower rates of service use, The British Association for Sexual Health and HIV (BASHH) Gender and Sexual Minority Special Interest Group produced expert recommendations for how SHS can provide inclusive care for T/GD people.^16^ These recommendations highlighted the lack of published evidence, including a need to understand service user perspectives. The aim of this study was to explore T/GD people’s experiences in SHS and perspectives on how to support their sexual health needs.

## Methods

### Design

This qualitative research was conducted by a cis-gender research team, guided by input from an expert steering group, consisting of representatives from trans community organisations and sexual health clinicians and from T/GD patient and public involvement (PPI) representatives. PPI representatives provided input on ethics submissions, reviewed interview and focus group discussion guides and provided feedback on the analysis. The project was also informed by published recommendations for research with trans participants.^1^ To support participation, participants were offered a choice of an interview or participating in a focus group and a voluntary demographic survey prioritised self-description. Ethical approval was granted by UCL Research Ethics Committee (8805/007).

### Participants and recruitment

Participants aged ≥16 years old who self-identified as transgender and/or non-binary and living in the UK were eligible to participate. In order to engage a population experiencing intersecting vulnerabilities, a combination of sampling strategies was employed and adapted iteratively during the study.^17^ Initially, adverts inviting potential participants to register their interest in the study were shared with community organisations and on social media (Twitter and Instagram). Subsequently, calls for participation were shared through informal networks via word of mouth and snowball sampling. Volunteers completed an online form providing their gender identity, pronouns and contact details. Non-responders were recontacted up to three times. Purposive sampling was used to ensure representation of different gender identities across the sample, to provide ethnic diversity and to include people who had recently used SHS. Participants gave informed consent to participate in the study and had the opportunity to ask questions of the researcher about the objectives of the study and his positionality before participating.

### Procedure

Interviews lasted 45–90 min and were conducted via Zoom by an experienced researcher (TW), guided by a topic guide (online supplemental document 1). Interviews explored participants’ experiences of accessing SHS and their reflections on the inclusivity of services. Focus groups lasted 90 min and were conducted via Zoom by TW, supported by a research assistant. Discussions focused on participants’ shared understanding of sexual health and reflections on an ‘ideal’ T/GD-inclusive SHS. Three patient vignettes, based on scenarios drawn from interviews, were used to stimulate discussion. Both interviews and focus group discussions were digitally recorded and transcribed verbatim. A voucher of £50 was offered to each participant. Data collection continued until the team judged that sufficient information power had been generated to meet the research aim, balancing the broad research question with the specificity of the population of interest and the richness of the interview and focus group data.^18^

### Analysis

Data were analysed by TW and LKM using thematic analysis,^19^ supported by NVivo. Interview and focus group transcripts were integrated and analysed together; the convergence of phenomena across the individual interview and group discussions was used to build the reliability of the analysis.^20^ Data were coded inductively and developed into initial themes. These themes were refined through iterative engagement with transcripts and named using data extracts from interviews and focus groups. Themes were validated by discussion between the research team (LKM, GR and JSa) and in consultation with steering group members (LH, LM and JSt) and PPI representatives.

## Results

**Figure 1:**
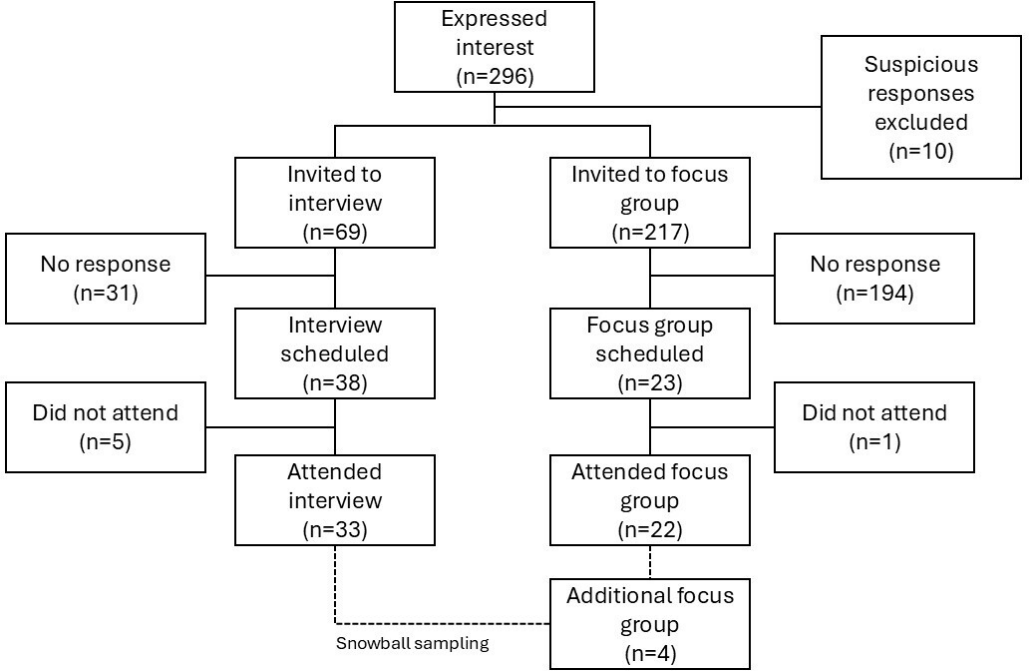
Participant enrolment.

**Figure 2:**
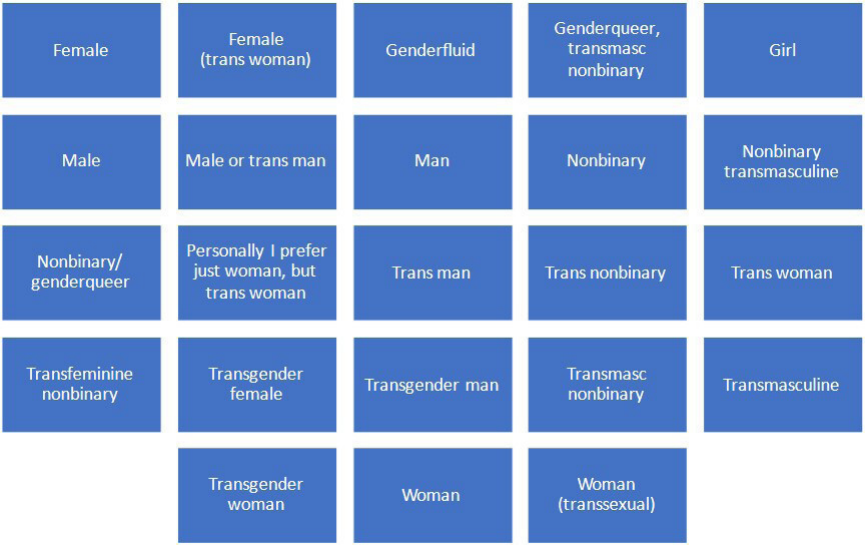
Participant self-described gender.

Two-hundred and ninety-six people expressed an interest in participating in the research, of whom 59 were recruited to the study (figure 1, table 1). Ten expressions of interest were flagged as suspicious based on similarities to reports of ‘imposter’ participants and were excluded.^21^ Thirty-three people participated in individual interviews. People who did not attend an interview did not provide feedback as to why they did not participate in the study. Three focus groups (n=8; n=8; n=6) were conducted online. Interviews and focus groups took place between May and July 2022. A low number of trans women/transfeminine people initially expressed an interest in participating. To address this, snowball sampling was used to recruit an additional focus group with trans women (n=4), which was conducted in July 2023. Participants self-described a diverse range of gender identities (see figure 2). Fifty-three participants provided additional demographic details, including age, ethnicity, sexuality, relationship status, education, employment, location and disability (see table 2).

**Table 1:** Participant summary—pronouns

Pronouns	n	%
He/him	18	30.5
She/her	16	27.1
Sie/hir/hirs	1	1.7%
She/they	1	1.7%
They/them	13	22.0%
They/he	4	6.8%
They/she	1	1.7%
They/them or he/him	3	5.1%
They/them or she/her	1	1.7%
They/them/xe/xem	1	1.7%
**Total**	**59**	

**Table 2:** Sociodemographic details (provided by 53 participants)

Age	n	%
17–24 years old	17	32.1
25–34 years old	24	45.3
35–44 years old	5	9.4
45–54 years old	5	9.4
55–64 years old	0	0
65 years or older	1	1.9
No answer	1	1.9
**Ethnicity**		
Black/black British	2	3.8
Indian/Indian British	3	5.7
Mixed	5	9.4
White/white British/white Irish/white Welsh	43	81.1
**Sexuality**		
Bisexual	14	26.4
Demisexual	3	5.7
Gay man	7	13.2
Gay woman/lesbian	3	5.7
Heterosexual	6	11.3
Pansexual	8	15.1
Queer	12	22.6
Current relationship*		
Dating	13	24.5
Engaged	3	5.7
Married/civil partnership	6	11.3
Monogamous	9	17.0
Open	7	13.2
Polyamorous	9	17.0
Separated/divorced	2	3.8
Single	17	32.1
**Education**		
No qualifications	1	1.9
GCSE or equivalent	4	7.5
A-levels or equivalent	11	20.8
Foundation degree or equivalent	4	7.5
Honours degree or equivalent	14	26.4
Masters degree or equivalent	15	28.3
Doctorate or equivalent	4	7.5
**Employment[Table-fn T2_FN1]**		
Employed full-time	24	45.3
Employed part-time	8	15.1
Employed flexibly	4	7.5
Full-time student	12	22.6
Part-time student	3	5.7
Not in education or employment	9	17.0
Retired	1	1.9
Self-employed	4	7.5
**Location**		
Urban (population>10 000 people)	45	84.9
Rural (population<10 000 people)	8	15.1
**Disability**		
Yes	26	49.1
No	27	50.9

* Total may exceed 53 and 100% as respondents could select more than one option.

Data were coded and organised into initial themes: barriers to inclusive care, facilitators of inclusivity and how participants characterised inclusivity. From these, three cross-cutting narrative themes and subthemes were developed, which are presented here. A summary of these themes and subthemes is provided in table 3 and supporting quotes in online supplemental document 2.

**Table 3:** Themes and illustrative quotes

Theme/subtheme	Description
***‘One way or another you’re not going to fit’***	
An expected lack of inclusivity	Participants expected services not to be inclusive or to be discriminatory
Poor experiences in other services	Experiences in other NHS services lowered participants’ expectations of care from sexual health services
Denial of care	Some participants reported that trans and gender diverse people are routinely denied sexual healthcare
‘Uphill struggle’	Participants described issues with being able to access interventions that are routinely offered to other demographics
The NHS doesn’t understand T/GD people	The lack of appropriate information led some participants to question if the NHS understood T/GD people
***‘If people come in, they shouldn’t be a surprise’***	
Specific T/GD inclusivity	In the context of increasing hostility towards T/GD people, participants sought indications that services would be specifically inclusive, such as a trans pride flag displayed in the service
Gender neutral greetings	Being greeted by staff in a gender-neutral manner made a significant different to participants
Physical spaces	The arrangement of physical space, including waiting rooms, and provision of gender-neutral toilets helped participants feel welcomed in services
Registration forms	Having inclusive options on forms helped build confidence that services were engaged with the needs of T/GD people
Sharing pronouns	Staff sharing their pronouns, or wearing pronoun badges gave T/GD service users confidence care would be inclusive
Service configuration	Participants expressed divergent opinions on whether services should be T/GD-specific or whether they would prefer inclusive general services and acknowledged that one size would not fit all
***‘You can talk to people normally’***	
Expectation of good care	Participants expressed a desire for all services—even non-specialised ones, to provide good quality, T/GD-inclusive care
Staff openness	Part of inclusive care for participants was staff having an openness towards T/GD service users’ needs and to learn from them
Educating staff	Participants were often ambivalent about educating staff about T/GD people’s sexual health needs
Specialisation	While participants did not expect all staff to have a detailed knowledge of T/GD people’s sexual health needs, they wanted access to expertise
Inappropriate curiosity	Participants experienced staff asking inappropriate questions about their identity that were not related to the provision of care
Inclusivity means being treated normally	For some participants, being treated carefully by staff was a source of frustration
Cis/heteronormative assumptions	Participants described how assumptions were made about their genitals and sexual practices when receiving sexual healthcare
Person-specific terminology	Having the opportunity to share the terms they preferred to use for their bodies was important for some participants

T/GD, trans and/or gender diverse.

### ‘One way or another, you are not going to fit’

Participants often believed that SHS were not set up for T/GD people. In some cases, this was based on negative experiences in other healthcare settings. Some who did engage with SHS described being denied care, either on the grounds of complexity, or because of ‘trans broken arm syndrome’^22^ where being transgender is inappropriately suspected as causing a complaint. Other participants portrayed an ‘uphill struggle’ accessing risk reduction interventions, such as HIV pre-exposure prophylaxis (PrEP).

Those who sought information about sexual health on the NHS website described how the lack of T/GD specific content meant the information was *aimed at the exact opposite people that I am* (Interview 1, she/her). Not only was this dehumanising and upsetting, it made some participants question whether the NHS acknowledges the existence of T/GD people. Participants also wondered if SHS essentialised T/GD service users as the sex they were assigned at birth, for example, that trans women are *people who have sex with men but who are, in the eyes of doctors, also kind of a man* (Focus group participant, she/her).

These experiences, both of SHS and wider healthcare, compounded by experiences of societal transphobia, contributed to low levels of engagement with SHS among participants and shaped the expectations of those who did engage with services.

### ‘If people come in, they shouldn’t be a surprise’

To overcome expectations of a lack of inclusivity, participants wanted to know that SHS had engaged with the needs of T/GD people. In the context of increasing hostility towards T/GD people, some sought reassurance that they would be welcome, *you don’t always have that assurance that [LGBT spaces] are going to be trans friendly* (Interview 11, she/her). Having Trans or Progress Pride flags in reception areas indicated active engagement with T/GD inclusivity. Some participants felt a statement of inclusivity was a sufficiently positive indicator but for others having explicit T/GD policies supported their trustworthiness, *describing on their website exactly the things that they do to make them trans-inclusive; not just saying that they’re trans-inclusive* (Interview 21, they/them/he/him).

Some had experienced being misgendered when arriving at SHS. In services where reception staff avoided using gendered terms when greeting people, this simple change had a positive impact. The arrangement of physical spaces and facilities was also important for a sense of being expected and welcomed by services, for example, not having waiting areas segregated by gender and provision of neutral toilets. Registration forms with appropriate gender options were also an indication of inclusivity and having non-binary options increased confidence that services were engaged with the needs of T/GD people. As well as indicating inclusivity, registration forms could also help facilitate consultations (see *You can talk to people normally*, below). Staff sharing their pronouns at the start of consultations or on name badges could help establish that they were engaged with gender diversity.

Participants had divergent opinions on how services should be configured. Some would prefer a dedicated clinic, feeling more comfortable in waiting areas with other T/GD people, but expressed concerns that this could make it a target for harassment or funding cuts. Others preferred being seen as part of general services, to support a sense of normality and to avoid ‘outing’ themselves.

### ‘You can just speak to people normally’

Interaction with staff during consultations was a key factor that underpinned good experiences of care. Anticipating having their identity questioned or needs dismissed, participants sought kindness and openness. Participants recognised that staff with less experience of providing care to T/GD people were anxious not to cause offence. While the majority appreciated the effort being made, some found it frustrating, *I get that it comes from a place from not wanting to upset me but… this conversation is just taking longer!* (Interview 27, he/him).

Participants emphasised the importance of not making assumptions about T/GD people’s genital configurations or sexual practices, for example, when providing kits for self-sampling or discussing event-based PrEP. They suggested that registration forms that gave service users the opportunity to share an ‘organ inventory’ could help facilitate this. Some participants had terminology they preferred to use for their bodies, for example, ‘front hole’ instead of ‘vagina’, and having the opportunity to share these preferences and have them used during consultations was affirming.

Participants did not expect healthcare professionals to have a detailed knowledge of T/GD people, but to have a general awareness of their sexual health needs. Although some felt it was important for them to teach clinicians about T/GD sexual health during consultations, others described situations where they had been made uncomfortable by intense questioning about topics not relevant to the consultation, for example, plans for affirming surgeries. Participants suggested that staff should be aware of and proactively offer additional services that would support T/GD people, including cervical smears for those with a cervix; monitoring for those self-sourcing gender-affirming hormones and T/GD appropriate support for people who had experienced sexual assault.

Although the specific needs of T/GD people might be diverse and different from cisgender service users, participants stressed that SHS staff already had the skills to deliver sensitive person-centred care: *you don’t have to build from the ground up in terms of knowing how to get people to talk about their bodies and their health problems* (Interview 17, he/him). Several participants who had positive experiences wanted to emphasise the value of inclusive SHS: *I feel very safe there. I feel listened to. And I don’t feel awkward or an anomaly or strange, or other. That’s really, really special in healthcare* (Interview 029, he/him).

## Discussion

Compounded by experiences of societal transphobia, participants’ negative experiences of healthcare contributed to low levels of engagement with SHS and shaped the expectations of those who did engage with services. This study found that T/GD people often expect that SHS are not designed to meet their needs and they look for signs when judging if a service is inclusive. It also found that a person-centred approach to consultations, along with basic awareness of T/GD people’s sexual health needs, can help to meet common expectations of good care. These findings highlight how simple changes can improve T/GD people’s experience of services. The lack of consensus among participants about whether they would prefer to access a T/GD-specific or generic SHS emphasises the need for T/GD-inclusive practice in all services.

The strengths of this research are its inclusion of participants with diverse gender identities and the involvement of a PPI group throughout the research. Through the development of narrative overarching themes, this analysis is specific to sexual health and goes beyond barriers and facilitators of access to healthcare, which have been well explored in other studies.^9 11^ By giving participants the opportunity to self-describe their gender, we highlight the range of terms people use and the limitations of applying traditional gender labels in research with T/GD populations. Making our demographic survey optional reduced barriers to participation but limits our ability to fully describe the sample. The data we do have highlight the predominantly white, urban, university educated nature of the sample (see table 2). This means that our findings likely underemphasise the role of intersecting inequalities. Conducting interviews and focus groups online also supported participation of a vulnerable and stigmatised population, affording participants options to control their identity, for example by using a pseudonym or keeping their camera off during research encounters.

The findings offer support for the recommendations of the BASHH Gender and Sexual Minority Specialist Interest Group regarding trans-inclusive SHS. The BASHH recommendations emphasise the need for gender-neutral registration forms, waiting and examination rooms as well as toilets. Additionally, they underscore the importance of staff undergoing equality and diversity training that includes specific information about the needs of T/GD service users. From a clinical perspective, the recommendations focus on several key aspects, including asking and respecting patients’ pronouns and tailoring STI testing to individual sexual practices and risks. The data in this study provide support from service users for these elements. In addition, the BASHH recommendations encourage inquiring about experiences of interpersonal domestic and sexual violence, recognising that these are common challenges faced by T/GD people.

The BASHH recommendations were published 4 years prior to this study being conducted and participants who had recently engaged with SHS often reported positive experiences of services. However, the widespread low expectations of SHS among participants suggest services can overcome these perceptions by actively promoting their inclusivity. The importance of community in engagement with healthcare has been stressed in other studies with T/GD people.^23^ This points to the importance of establishing the trustworthiness of services as part of this process. Research on physician–patient relationships point to the role that social and historical context can play in mistrust between people from marginalised groups and healthcare services as well as the importance of respect, partnership and time and consistency in building trust.^24 25^ Other studies of LGBTQ+experience of services describe how physical spaces, service infrastructure and interactions with staff are all critical to creating ‘safe spaces that matter’.^26^ Allyship for healthcare professionals includes embracing new ways of delivering care to those who might typically be excluded and aligns closely with key principles of person centeredness, patient advocacy, individual autonomy and social justice.^27^ Τ/GD people in this research had diverse sexual health needs, linked to not only their identity and gender affirming care but also their relationships and sexual practices, which changed over time for many. However, by following a person-centred approach to consultations, the needs and priorities of each individual can be ascertained and met. National guidelines on person-centred sexual history taking were updated to reflect gender diversity in 2019.^28^

These findings help to provide an evidence base for the BASHH expert recommendations and to provide clinicians with some very simple and easy to adopt recommendations that make a significant impact on T/GD service users, such as avoiding gendered language in greetings and including pronouns when introducing themselves. They also outline structural changes that could be made to facilitate easier interactions, for example, on registration forms, electronic patient records and self-sampling kits. However, they also highlight the challenges of providing services that will meet everyone’s preferences but provide principles of inclusivity that can be delivered in all services.

Future research on provider perspectives in UK SHS could complement this research, identify areas of inconsistency between provider and service user needs and be used to update recommendations for services and consultations. They could also inform the development of further research to quantify how common the experiences described in this research are and develop a more nuanced understanding of how they are associated with sociodemographic and behavioural variables.

## Conclusion

These findings support the BASHH GSM expert recommendations for inclusive SHS. They highlight the need for services to actively engage to reach a population with potentially greater sexual health needs who are disengaged with services. To meet the needs of range of T/GD people, there is a need for both specialised clinics and general inclusivity of services.

## Supplementary material

10.1136/sextrans-2024-056231 online supplemental file 1

10.1136/sextrans-2024-056231 online supplemental file 2

## Data Availability

No data are available.
