# Supplementary material for: One way or another, you are not going to fit: trans and gender diverse people’s perspectives on sexual health services in the United Kingdom
Source: Sex Transm Infect. 2025 Jan 20;101(5):e056231. doi: 10.1136/sextrans-2024-056231 (PMC12322470; doi:10.1136/sextrans-2024-056231)
Supplement: online supplemental file 1 [file sextrans-101-5-s001.docx]

**INTERVIEW TOPIC GUIDE**

- Check participant comfortable, quiet, secure location
- Check time available
- Agree procedure on breaks, connectivity issues
- Consent documented
- Reiterate anonymity – nothing you say to me will be linked back to you
- Reiterate ability to withdraw – some questions may be sensitive; you don’t have to tell me anything that makes you uncomfortable
- Signpost support organisations - support available - and provide list
- Final questions?

Informal interview, interested in your experiences... three sections, first about sexual health in general, then more specifically about your sexual health and finally about your experiences of sexual health services

1. Can you start by telling me how you found out about this study?
   - *Prompts:* Where saw advertised, personal recommendations
   - *Probe:* Why did you want to take part?
   - *Probe:* What do you want to get from participating?
2. When you think of sexual health, what do you think of?
   - *Prompts*: having pleasurable sex, STIs, HIV, having choices, freedom, positive self-esteem, health body, healthy mind, sexual function, sexual experiences free of discrimination/violence.
   - *Probes*: What aspects are most important to you?
3. Lots of people use the term ‘safer sex’. What does that term mean to you?
   - *Probe:* Has your understanding of what is safer sex changed?
   - *Probe:* What influenced these changes to your understanding?
4. Can you give me an example of a time when you wanted to find information about sexual health?
   - *Prompts*: Where did you go to find information: Sexual health service, GP, gender clinic, internet, friends, family, school, charity.
   - *Probes*: What did you think of this/those experiences?

In the next part of the interview, I will be asking questions that will help me to gain a better idea of what your sex life is like for you these days, and how you feel about it. Reminder that you don’t have to tell me anything that makes you uncomfortable

1. Tell me about your sex life in general...how is it going for you?

• *Probes*: In specific ways / circumstances?

• *Probes*: What is going well? What could go better?

1. Are you (or have you ever been) concerned about your sexual health?
   - - *Prompts:* HIV, STIs, contraception?
     - *Probes:* Can you tell me more about that? What concerned you/what didn’t?
     - *Probe:* What did you do because of that concern?
     - *Probes:* What did you find helpful? What could have been done to help your concern/make the process easier?
2. Where do you go to for advice on, or decisions around, your sexual health?
   - *Prompts*: Contraception, HIV/STI testing and prevention, sexual difficulties.
   - *Prompts*: Sexual or intimate partners, friends, family, internet, healthcare professionals, gender clinic, charity.

In this last part of the interview, I will be asking questions about your experiences – both positive and negative – of using sexual health services, either on-line, in person, through the NHS, charities or in any other way

1. Can you tell me about your experiences accessing sexual health services? (If any)
   - *Prompts*: Going to a SHS, GP, gender clinic, pharmacist, online, charity
   - *Prompts*: HIV/STI prevention (testing, treatment), PrEP, PEP, contraception, smear, pleasure
   - *Prompts*: Finding information, getting an appointment, at the clinic
   - *Probes*: Can you tell me a bit more about that experience?
   - *Probes*: Have you experienced any barriers to accessing services?
   - *Probes:* What made/could have made it difficult? What made/could make it easier?
2. Have you ever experienced any negative interactions whilst using sexual health services?
   - *Probes:* Can you tell me a bit more about that? How did that make you feel?
   - *Probe:* What could have been done differently?

1. Have you ever experienced any positive interactions whilst using sexual health services?
   - *Probes:* Can you tell me a bit more about that? How did that make you feel?
   - *Probe:* What was it about the interaction that gave you a positive reaction?
2. Has your gender identity ever come up in a conversation with a sexual health clinician?
   - *Probes:* How did it come about? How did the conversation go? How did that make you feel?
   - If not, how would you feel about discussing your gender identity with a sexual health clinician?
3. If you have accessed a gender clinic, has your sexual health ever come up in a conversation with a gender clinician?
   - *Probes:* How did it come about? How did the conversation go? How did that make you feel?
   - If not, how would you feel about discussing your sexual health with a gender clinician?
4. What does an inclusive sexual health service mean to you?
   - *Probe:* What would distinguish an inclusive service from a non-inclusive one?
5. Is there anything final you would like to add or share that we have not touched on?

**Thank you. Confirm email address to send voucher**
