# Supplementary material for: One way or another, you are not going to fit: trans and gender diverse people’s perspectives on sexual health services in the United Kingdom
Source: Sex Transm Infect. 2025 Jan 20;101(5):e056231. doi: 10.1136/sextrans-2024-056231 (PMC12322470; doi:10.1136/sextrans-2024-056231)
Supplement: online supplemental file 2 [file sextrans-101-5-s002.docx]

**Online supplementary document 2: Themes and illustrative quotes**

| Theme/subtheme | Description | Quotes |
| --- | --- | --- |
| ***“One way or another you’re not going to fit”*** | | |
| An expected lack of inclusivity | Participants expected services not to be inclusive or to be discriminatory | “Services have inherently cis-normative, hetero-normative assumptions […] obviously as a trans person you are most likely in one way or another - by virtue of being trans - not going to fit”  Focus group participant (they/he) |
|  |  | “It’s scary by definition. The fear is that you are going to be treated badly. You’re going to be treated as something different rather than just an individual with those concerns, those problems, you know”  Interview 14 (she/her) |
| Poor experiences in other services | Experiences in other NHS services lowered participants’ expectations of care from sexual health services | “I think the thing about trans healthcare in this country that is necessary to understand as a first principle, is that everything is on fire […] The assumption facing a trans person from the moment they enter the system as a patient, is that they are somehow a problem”  Interview 17 (he/him) |
|  |  | “[Waiting a long time for gender services] does colour how you see the rest of the NHS, because you’re getting the message that nobody cares about me, it’s fine for me to wait five years for care, for something that’s really important to me. So you do tend to think that the rest of the NHS will treat you like shit as well, and have a long waiting list, misgender you all the time. It doesn’t improve patient relations with sexual health services, or anybody else”  Interview 20 (he/him) |
|  |  | “It’s not a secret that no one in this country knows fucking anything about trans healthcare because it’s literally the biggest fucking mess. So when it comes to trans stuff, a healthcare provider is a means to an end. I’m not always honest with them; I will do what I have to do to get the help I need from them and I don’t expect them to be, necessarily, on my side in seeking that help”  Interview 25 (they/he) |
| Denial of care | Some participants reported that trans and gender diverse people are routinely denied sexual health care | “If we disclose our trans status when seeking sexual healthcare, we will immediately be denied, like before anything else. It’s either, ‘You’re too complicated to deal with, you need a specialist,’ or ‘Hmm, yes, you do have raging thrush and do you think that that’s probably because of your hormones and you should de-transition about that.’”  Interview 17 (he/him) |
| “Uphill struggle” | Participants described issues with being able to access interventions that are routinely offered to other demographics | “I had a great deal of trouble accessing PrEP because they don’t offer PrEP to heterosexual cisgender women and it took me quite a while to convince them, on the phone, that that was not me… the service is not accessible in that way unless you fit their expectations.”  Interview 24 (she/her) |
| The NHS doesn’t understand T/GD people | The lack of appropriate information led some participants to question if the NHS understood T/GD people | “It’s very ‘these are the body parts a man has, and these are body parts a woman has.’ And if you’re trans you’re going to have to ask for something different”  Interview 4 (he/him) |
|  |  | “Any time I want to look up something [on the NHS website] related to body parts I have it’s always “women sometimes blah, blah”, and I have to actively push past that to absorb the information when it’s misgendering me as I’m reading. […] There are days when I genuinely don’t care but other days it does impede me processing the information when I have to read the word woman in every paragraph”  Interview 28 (he/him/they/them) |
| ***“If people come in, they shouldn’t be a surprise”*** | | |
| Specific T/GD inclusivity | In the context of increasing hostility towards T/GD people, participants sought indications that services would be specifically inclusive, such as a trans pride flag displayed in the service | “It’s nice to see [the trans pride flag] but you see it and you think, OK, this organisation probably isn’t going to be transphobic. And, I think just people being explicit about their trans inclusivity because, sadly, you know, simply having a generic Pride flag or saying we’re LGBT supportive, that’s not really the comfort anymore that it necessarily should be.”  Interview 12 (she/her) |
|  |  | “The flag always helps but not just the generic rainbow flag because we know the NHS have taken that and that makes it ambiguous but the Progress flag, or the trans flag. Or something like it being visibly there on the website, or saying we cater to LGBT people or something like that, just making it very specifically clear that it is friendly.”  Interview 29 (he/him) |
|  |  | “Having a trans flag, because you can see, at least they’re doing a little bit. So they’ve thought about it as opposed to not having thought about it at all.”  Interview 04 (he/him) |
| Gender neutral greetings | Being greeted by staff in a gender-neutral manner made a significant different to participants | “That’s the difference between me feeling comfortable and uncomfortable. I don’t think the average man or woman is bothered whether they get called sir or madam at a clinic. Just say, hello, that’s it.”  Interview 3 (she/her) |
| Physical spaces | The arrangement of physical space, including waiting rooms, and provision of gender-neutral toilets helped participants feel welcomed in services | “Oh my god, they had like women over there, and men over there… and I don’t know what I would do now if I was going to that clinic like being trans”  Interview 20 (he/him) |
|  |  | “With toilets, you know, inaccessibility to these toilets, kind of, going into a service and it has a men’s and a women’s, and it’s like already you’re fucking things up a little bit here.”  Interview 11 (she/her) |
| Registration forms | Having inclusive options on forms helped build confidence that services were engaged with the needs of T/GD people | “Looking at a form and is it non-binary inclusive, because even though I’m not non-binary if I know that they’re being non-binary inclusive I know that they’ve thought about trans men as well”  Interview 04 (he/him) |
| Sharing pronouns | Staff sharing their pronouns, or wearing pronoun badges gave T/GD service users confidence care would be inclusive | “I find [it] easier when the other person introduces their pronouns. That makes that ‘OK now I can’ because I often am scared that these health providers will have their own biases”  Focus group participant (they/them) |
| Service configuration | Participants expressed divergent opinions on whether services should be T/GD-specific or whether they would prefer inclusive general services and acknowledged that one size would not fit all | “Having specific clinic times set aside for trans-people so that you’re not waiting in a room and worried about other people looking at you and going ‘who’s that?’”  Interview 21 (they/them/he/him) |
|  |  | “Essentially, I’m with like-minded people […] OK no one in this waiting room is looking at me as if I’m a freak of nature walking through the door, which just makes the whole process just feel a little bit nicer”  Interview 11 (she/her) |
|  |  | “In an ideal world, everything is just sexual health care and it's normalised for everyone. If you have more specialised [T/GD] clinics, maybe you have some lovely people lurking outside that want to harass anyone that goes in”  Focus group participant (she/her) |
|  |  | “You know, how insane the wait list for the GIC [gender identity clinic] is, [T/GD specific clinics] could just end up being a repeat of that”  Focus group participant (she/her) |
|  |  | “It would just be nice if things that are good for trans people didn’t have to be so kind of portioned off from everything else […] you know, 10 years ago I wouldn’t go into a waiting room if I knew that it meant that being sat in there meant that everyone in there knew I was trans… I think when people try and be accessible they need to consider that that in itself is actually a hurdle for some people and that it’s good to kind of mix things up a bit so, that there’s options for everyone”  Interview 27 (he/him) |
| ***“You can talk to people normally”*** | | |
| Expectation of good care | Participants expressed a desire for all services – even non-specialised ones, to provide good quality, T/GD-inclusive care | “Just having confidence that we’re going to get the same sort of treatment and good treatment from whoever that it is that we approach because everyone is confused, and that’s not good.”  Focus group participant (he/him) |
|  |  | “It would just be nice if things that are good for trans people didn’t have to be so kind of portioned off from everything else”  Interview 27 (he/him) |
| Staff openness | Part of inclusive care for participants was staff having an openness towards T/GD service users’ needs and to learn from them | “It’s very much about just doctors in general how they present themselves to you, you’re more likely to respond if they seem open and not like they’re going to judge you”  Interview 6 (they/he) |
|  |  | “Just that you have that connection with [staff] – you can just relax in the situation and you don’t feel they’re going to find you odd, or not really understand you, or you’re just not on the same page […] And an openness, like a curiosity but not like an over curiosity but like… kindness and understanding”  Focus group participant (they/he) |
| Educating staff | Participants were often ambivalent about educating staff about T/GD people’s sexual health needs | “It’s unfortunate that it’s trans people attending these services that have to teach the service providers how to do their job properly, but sometimes that is how it goes.” Interview 18 (he/him) |
|  |  | “I am patient with people because they’re not asking out of malice, they generally genuinely want to know… The harm has already been done to me, if I educate these people now it means that the next person who comes in and is in my position isn’t made as uncomfortable”  Focus group participant (they/them) |
| Specialisation | While participants did not expect all staff to have a detailed knowledge of T/GD people’s sexual health needs, they wanted access to expertise | “I don’t expect every sexual health professional to be fully up to date with all the latest trans-specific stuff, I know that we’re a niche group. But for there to be some very explicit ones to go to, maybe if a certain clinic has a worker on staff who maybe specialises in this sort of thing” Interview 03 (she/her) |
|  |  | “I’m sure there are some really good professionals out there when it comes to sexual health and gender diverse people. But I think you would still rather start with someone you know gets it”  Interview 03 (she/her) |
| Inappropriate curiosity | Participants experienced staff asking inappropriate questions about their identity that were not related to the provision of care | “Last time I was in for a sexual health check-up, the person who was asking me all these questions about what surgeries I had had, which is fair enough I can excuse that because that might be relevant to later on in the consultation or whatever, but then was asking me all these questions about what surgeries I have got planned to have, and what’s down the road for me, and why is it hard for trans people to come to sexual health clinics, and why does that cause dysphoria and why does that make you uncomfortable and how did you know that you were trans, and when did you find out, and how old were you? And that’s pushing the professional role too far.”  Focus group participant (they/them) |
| Inclusivity means being treated normally | For some participants, being treated carefully by staff was a source of frustration | “I guess it’s trying to remember that you can kind of just speak to people normally […] I get that it comes from a place from not wanting to upset me but then I almost find it frustrating because I’m like… this conversation is just taking longer!”  Interview 27 (he/him) |
| Cis/heteronormative assumptions | Participants described how assumptions were made about their genitals and sexual practices when receiving sexual healthcare | “If I put in that I was a man and that I had sex with men I would get a throat swab, a bum swab, and a pot to piss in – there was no way for me to communicate that I needed the [vaginal] swab not the pot… eventually they’d figure it out… and they would just hand me back a single swab…”  Interview 27 (he/him) |
|  |  | “When I started on PrEP, they were like, “You can do the event-based PrEP”, and then decided that I couldn’t […] based on nothing other than their own preconceived notions of how they think that a trans person of my type should have sex”  Focus group participant (they/them) |
|  |  | “Things can be a bit one size fits all unless you ask for it to be different […] when actually what they could do is be asking about body parts. So, these are questions we ask everybody which of these types of sex do you have […] So there’s ways of asking it, do you have sex with people with penis, people with vagina, you know, doing it that way, more like an inventory. And then from there that could inform what labels get printed out for you or what testing kit gets sent to you, rather than just these very, sort of, non-binary exclusionary quite frequently” Interview 04 (he/him) |
| Person-specific terminology | Having the opportunity to share the terms they preferred to use for their bodies was important for some participants | “If you’re doing like the list the contents of your abdomen thing you can also just put next to it like preferred language for this thing, so just have a little write in… Please don’t refer to this as a vagina, please just say this”  Focus group participant (he/him) |
|  |  | “I don’t like to use female terminology for my body so I would say I have frontal sex or the front hole or instead of the term vagina, I really hate that word it makes me feel uncomfortable”  Interview 29 (he/him) |
